# Numerical Simulation and Comparison of the Mechanical Behavior of Toughened Epoxy Resin by Different Nanoparticles

**DOI:** 10.1021/acsomega.3c03093

**Published:** 2023-08-15

**Authors:** Binbin Zhao, Yiqiao Zhao, Yiou Shen, Haoran He, Zehua Qu

**Affiliations:** † School of Aerospace Engineering and Applied Mechanics, Tongji University, Shanghai 200092, China; ‡ State Key Laboratory of Molecular Engineering of Polymers, Department of Macromolecular Science, Fudan University, Shanghai 200092, China

## Abstract

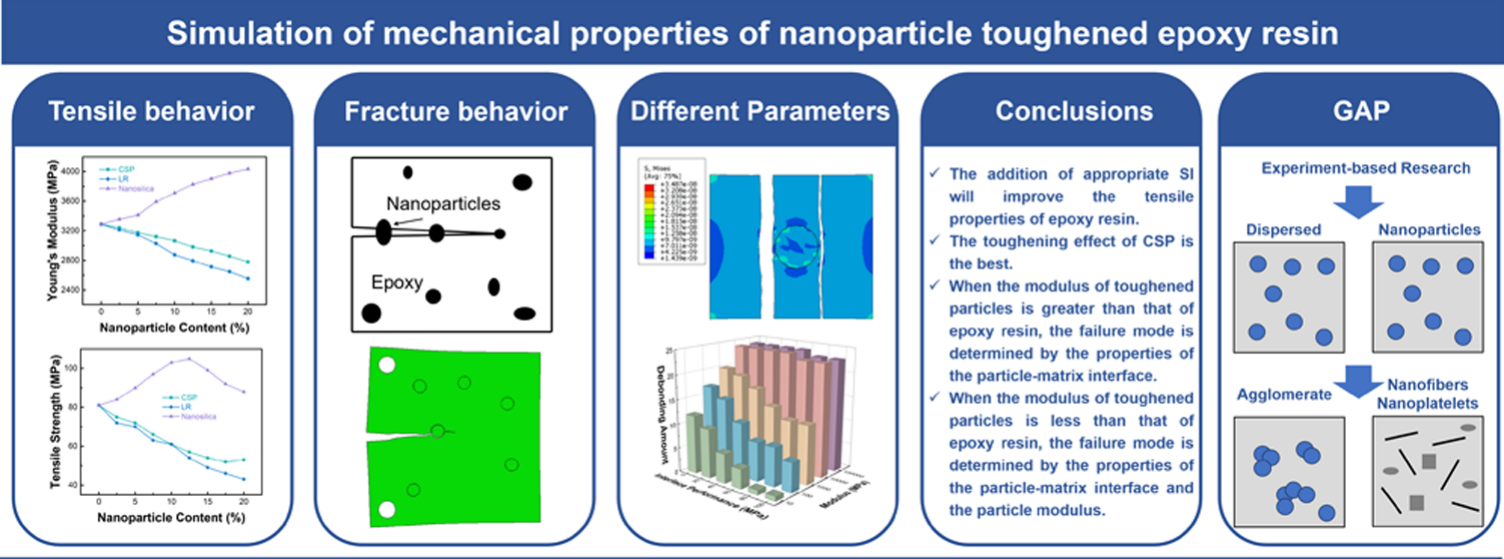

Adding nanoparticles as the second phase to epoxy can achieve a good toughening effect. The aim of this paper is to simulate the toughening behavior of epoxy resin by different nanoparticles using a convenient and effective finite element method. The mechanical behaviors of epoxy resins toughened by nano core–shell polymers, liquid rubber, and nanosilica were compared by numerical simulations using the representative volume element (RVE). It is indicated that the addition of a nano core–shell polymer and liquid rubber can reduce the tensile properties of epoxy resin, while nanosilica is on the contrary. With the increase of nanoparticle content, the length of crack propagation decreases, and the toughening effect of the nano core–shell polymer is the best. The failure mode is determined by the particle/matrix interface when the modulus of the nanoparticle is much larger than that of epoxy resin. However, it is determined by the interface properties of the particle/matrix and the modulus of nanoparticles in other cases. The results provide a theoretical basis for toughening nanoparticle selection of nanoparticle-toughened epoxy resin and other similar simulations in the future.

## 1 Introduction

Epoxy resins are widely used as matrices in fiber-reinforced composite materials due to their good processability, adhesion, low shrinkage, high chemical stability, and low water absorption.^1−6^ Epoxy resins reinforced by fibers have a series of advantages, such as lightweight, high specific strength, high specific modulus, and so on.^7−12^ However, after curing, the epoxy resin forms a cross-linked network structure with high brittleness, poor impact resistance, and insufficient crack propagation resistance.^13−15^ These shortcomings not only affect the impact properties and fracture toughness of fiber-reinforced epoxy resin matrix composites but also restrict the application of epoxy resin matrix composites.^16^ The common methods of toughening epoxy resin include nanoparticle toughening, thermoplastic resin toughening, interpenetrating network structure toughening, chemical network structure changing toughening, and so on.^17,18^ Different toughening methods have different toughening mechanisms, single action, or synergistic effects. Nanoparticle toughening could significantly improve the toughness of the epoxy resin. Nanoparticles commonly used in toughened epoxy resin include nano core–shell polymers, liquid rubber, nanosilica, carbon nanotubes, clay, etc.^19−23^

Adding liquid rubber is one of the earliest ways to toughen epoxy resins.^22^ The main toughening mechanism of liquid rubber is the local shear yield of the matrix around the particles and the anchoring mechanism. The experimental results show that there are holes in the liquid rubber particles or at the interface of the matrix and the particles during loading. These holes can reduce the hydrostatic stress at the crack tip and promote a large range of local shear yield.^24^ Nano core–shell polymer particles are another kind of special rubber toughening particles commonly used. The shell and core are made of different materials; the shell is generally harder and the core is softer. The toughening mechanism of nano core–shell polymers is similar to that of liquid rubber. In the case of no agglomeration of nano core–shell polymers (Tsang et al.’s experiment showed that agglomeration of nano core–shell polymers would significantly reduce the toughening effect^25^), the typical experimental results were reduced modulus and significantly increased fracture strength and energy.^26−29^ Quan et al.^29^ showed that the toughening effect was enhanced with the increase of the content of the nano core–shell polymer in epoxy within a certain concentration range and then entered a region where the toughening effect remained unchanged. Different from rubber particles, there are two toughening mechanisms in epoxy resin toughened by nanosilica: (1) The debonding of silicon particles followed by the growth of plastic cavities. The release of triaxial stress leads to the debonding of silicon particles, resulting in shear yield. (2) The local shear yield around the silicon particles.^22^ Ma et al. found that the influence mechanism of particle size on toughening effect is complex; the toughening effect of nanosilica particles with a diameter of 20 nm is better when the particle volume fraction is smaller. The toughening effect of silica nanoparticles with a diameter of 80 nm is better when the particle volume fraction is larger.^30^ Nanosilica particles with a diameter of about 20 nm are generally used for epoxy toughening.^31,32^

Most of the existing research on toughening epoxy resin with nanoparticles is carried out on macro specimens. The conclusion is obtained by observing the experimental data and the scanning electron microscopy images of the cross section and combining them with the theoretical calculation formula.^33^ For epoxy resin toughened by nanoparticles, the deficiency of experimental research is that the workload is huge, and the microscopic damage process of the nanoparticle-toughened epoxy resin system cannot be directly observed. However, there are few simulation studies on this aspect. Wang et al.^34^ studied the influence of core–shell nanoparticles on the yield behavior of epoxy resins by using representative volume elements, which was in good agreement with the yield function. Lin et al.^35^ established a model of the epoxy resin nanocomposite modified by boron nitride@boronate to analyze the fracture mechanism combined with experiments. Wang et al.^36^ used the extended finite element method to simulate the dis-adhesion of nanosilicon particles in epoxy resin and matrix crack growth and determined the weak interface toughening and strengthening mechanism of nanocomposite materials through the simulation results. Overall, the simulation study of the toughening of nanoparticle epoxy resin is limited and incomplete. It is difficult to understand the toughening mechanism of epoxy resin by different nanoparticles on the microscopic damage level.

In order to establish a simple and effective simulation method to analyze the toughening mechanism from the microscopic level, systematically compare the toughening effect of different nanoparticles, and provide a theoretical basis for the selection of toughening nanoparticles, we used a representative volume element model to study the tensile model of single nanoparticles, the toughening effect of different nanoparticle contents, and the influence of different nanoparticle parameters on the toughening effect.

## 2 Methods

### 2.1 Establishment of the Finite Element Model

#### 2.1.1 Scale and Units of the Finite Element Model

In this paper, commercial finite element software ABAQUS is used for numerical simulations. Considering the difference in the diameters and densities of the three kinds of nanoparticles (nano core–shell polymer, liquid rubber, and nanosilica, hereinafter referred to as CSP, LR, and SI, respectively), the modeling based on the volume fraction is more convenient and accurate.^37,38^ Referring to the existing experimental and theoretical studies, the particle size of CSP and LR is set as 300 nm,^25^ and an appropriate model size is 4000 nm × 4000 nm, while the particle size of SI is 30 nm,^30^ and the model size is 400 nm × 400 nm. The minimum size of the model in this software is not less than 10^–5^. Except for Poisson’s ratio, which is a dimensionless physical quantity, other physical quantities need to be converted, as shown in Table 1.

**Table 1:** Unit System of Three Types of Nanoparticles

parameters	nano core–shell	liquid rubber	nanosilica
length (nm)	10	10	1
modulus (Pa)	10^10^	10^10^	10^12^
Stress (Pa)			
fracture toughness (J/m^2^)	10^8^	10^8^	10^9^
element stiffness (N/mm^3^)	10^15^	10^15^	10^18^

#### 2.1.2 Representative Volume Element (RVE)

**Figure 1:**
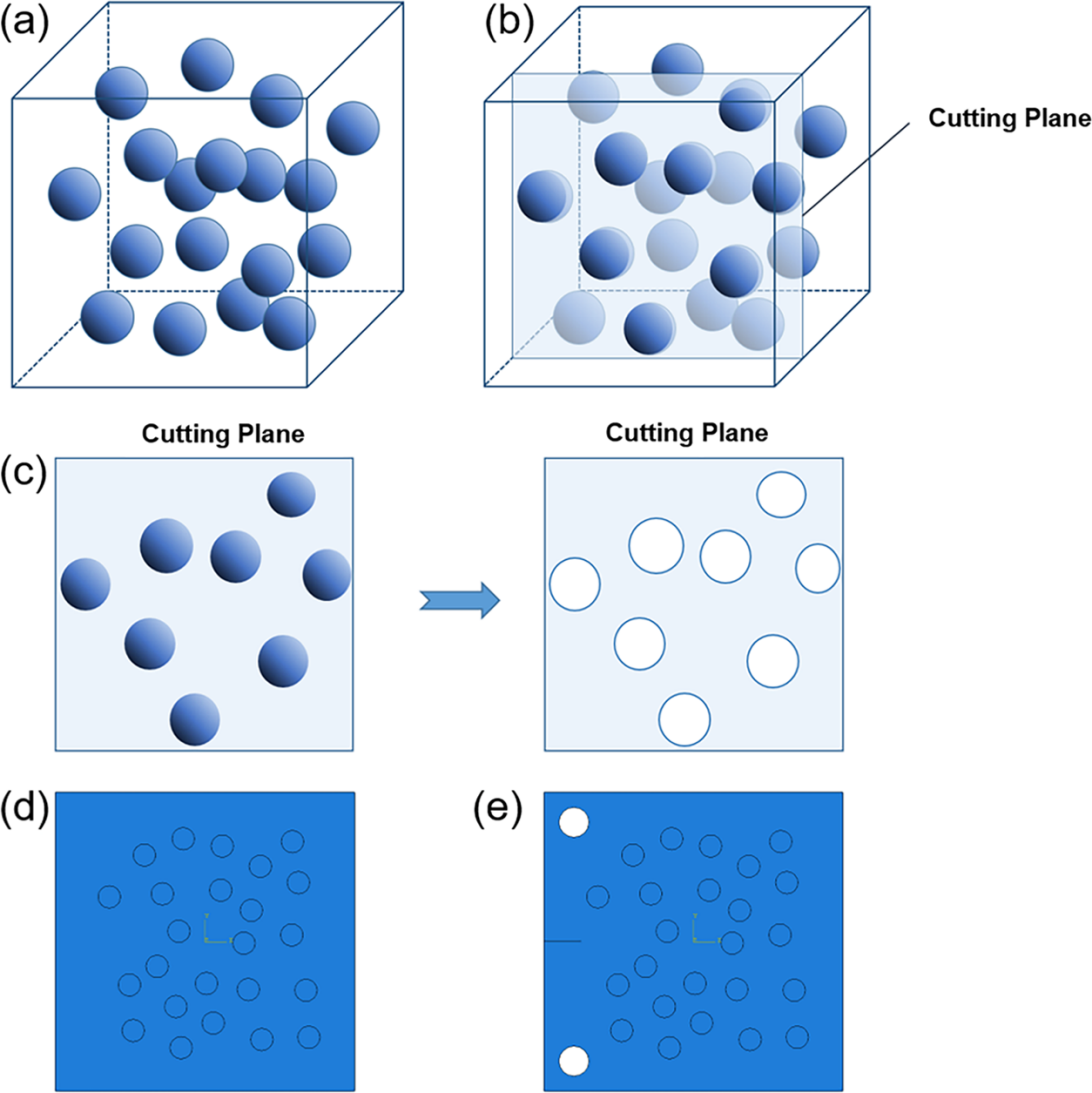
Selection of the representative volume element. (a) 3D representative volume element with random distribution. (b) Section selected from 3D representative elements. (c) 2D plane model RVE. (d) The 2D tensile model with a 20% volume fraction. (e) The 2D fracture model with a 20% volume fraction.

The numerical simulation of the microscale generally takes the representative volume element as the research object, and the reasonable selection is beneficial to reduce the calculation cost and present the failure mechanism more clearly. For the research object of this topic, there are two choices of representative volume element available: regular distribution representative volume element^34^ and random distribution representative volume element.^39^ In practice, even if the particle agglomeration phenomenon is not considered, the particle distribution in the matrix cannot be completely uniform but closer to the random distribution. Therefore, as shown in Figure 1a random distribution representative volume element is selected for this subject. In order to simplify the element calculation, a two-dimensional section (Figure 1b) is selected from the representative volume element of the three-dimensional cube to convert the three-dimensional model into a two-dimensional model, which was proved feasible by Wang et al.^36^ Despite the particle region having different sizes at different cross sections, it is found that the particle area with a smaller diameter on the cross section has little effect on the performance of the matrix, and the influence mechanism of different size particle areas on the surrounding matrix is similar in preliminary simulations. To sum up, the representative volume element can be set as a two-dimensional plane model with particles of the same size (Figure 1c).

In the process of modeling, the positions of particles are randomly generated, and then the combination of particle positions that can make particles evenly distributed is selected. The positions of particles in the model are adjusted not too close to the model boundary in order to avoid causing obvious stress concentration. Figure 1d,e shows the tensile mode and fracture model of the two-dimensional model with a 20% volume fraction.

### 2.2 Material Parameters of the Finite Element Model

By the Halpin–Tsai semiempirical model,^40^ Young’s modulus of the core–shell polymer can be calculated as follows
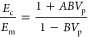
1
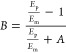
2Here, *E*_m_ is Young’s modulus of the epoxy matrix, *E*_c_ is Young’s modulus of modified epoxy resin. *E*_p_ is Young’s modulus of particles. *V*_p_ is the volume fraction of particles. *A* is the particle shape factor; for round particles, *A* = 2. *B* is the parameter related to Young’s modulus of particles and the matrix.

According to the research conclusion of Bucknall et al.,^41^ the bulk modulus of liquid rubber is about 2 GPa, and Poisson’s ratio is close to 0.5. The bulk modulus formula is as follows
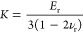
3Here, *K* is the bulk modulus formula of the liquid rubber toughened epoxy resin, *E*_r_ is Young’s modulus, and *ν*_r_ is Poisson’s ratio of liquid rubber. Gunwant et al.^42^ set Poisson’s ratio as 0.49992 in the numerical simulation study on toughening of liquid rubber. However, the maximum Poisson’s ratio in Abaqus can only be 0.495. In order to achieve a volume modulus of 2 GPa, Young’s modulus of liquid rubber particles is set to 60 MPa.

In the numerical simulation, the interface properties of the particle and matrix are determined by the separation initiation condition and separation expansion condition of the cohesive element. The criterion of maximum principal stress is selected; that is, when the von Mises stress on the cohesive element reaches the maximum value, the element begins to separate. The BK rule in the energy criterion is selected as the separation and expansion conditions, and the energy release rate should be set during the separation and expansion. The principle is elaborated in the following part. The initial stress of separation was set to 40 MPa, and the energy release rate was set to 100 J/m^2^ in the model of LR and CSP. The initial stress of separation is set to 1 MPa, and the energy release rate during separation and expansion is set to 5 J/m^2^ in the model of SI. In addition, the cohesive element stiffness is set to 10^4^ N/mm^3^ in the study.^43^ Young’s modulus and Poisson’s ratio of epoxy resin, CSP, LR, and SI are given in Table 2.^29,36,40,41^Table 3 lists the parameters of interface performance.^43^

**Table 2:** Young’s Modulus and Poisson’s Ratio of Epoxy Resin, CSP, LR, and SI^29,36,40,41^

material	epoxy resin	nano core–shell	liquid rubber	nanosilica
modulus	3290 MPa	775 MPa	60 MPa	70 GPa
Poisson’s ratio	0.35	0.4	0.495	0.16

**Table 3:** Parameters of Interface Performance^43^

polymer Model	CSP	LR	SI
energy release rate (J/m^2^)	100	100	5
initial separation stress (MPa)	40	40	1
stiffness of the cohesive element (N/mm^3^)	10^4^		

### 2.3 Method of Mesh

**Figure 2:**
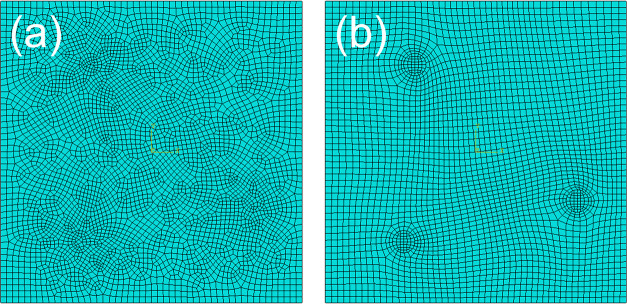
Results from different mesh generation methods: (a) advancing front method and (b) medial axis method.

Different numbers of grids will affect the calculation results. To determine the optimal grid number, by calculating the ratio of the von Mises stress in the stress concentration region to the model average,^42^ it is found that the stress concentration coefficient around the particle area almost does not change when the number of grids reaches more than 2700, and increasing the number of mesh at this time will only lead to a larger amount of calculation. Therefore, a model with a particle volume fraction of 2.5% is taken as an example (Table 4). Considering that the particle–matrix boundary is circular, using the medial axis method (Figure 2b) can obtain a more orderly unit arrangement than the advancing front method (Figure 2a).

**Table 4:** Relationship between the Stress Concentration Factor in the Particle Area and the Number of Grids in the Model with a 2.5% Particle Volume Fraction

grid size of the matrix	grid size of particle–matrix boundary	grid number	stress concentration factor
20	15	465	1.45
20	10	503	1.87
15	10	768	1.94
15	5	1043	2.05
10	5	1870	2.15
8	5	2711	2.15
8	3	5792	2.17
5	3	6942	2.18
5	2	8171	2.18

### 2.4 Finite Element Calculation Method and Principle

#### 2.4.1 Principle of the Cohesive Model

**Figure 3:**
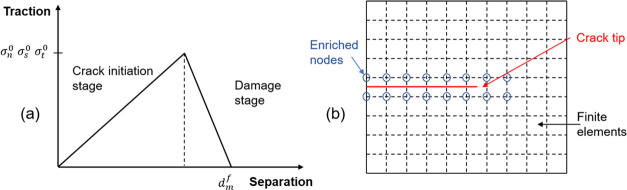
Schematic diagrams of finite element calculation. (a) Relationship between traction and separation in the damage process of the cohesive element. (b) Crack passes through elements.

The cohesion model simulates cracks by presetting crack edges (two-dimensional model) or surfaces (three-dimensional model). The elements used in the cohesive model are called cohesive elements. The cohesive model is simulated by inserting a layer of cohesive elements with a thickness of 0 in the preset crack area. The simulation of the damage process by the cohesive model is represented by the traction–separation theorem, which describes the relationship between traction and displacement on a macro level. The commonly used linear elastic model is shown in Figure 3a. In the crack initiation stage, the traction force increases with an increase in displacement. In the damage stage, the traction decreases with an increase in displacement.

The stress–strain relationship before damage is as follows
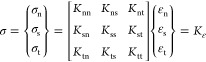
4Here, σ, *K*, and ε are the stress, stiffness matrix, and strain, respectively.
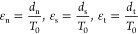
5Here, *T*_0_ is the original thickness of the cohesive element. The damage model consists of the damage initiation criterion and the damage evolution law. When the damage initiation criterion is reached, damage will then be carried out according to the defined damage evolution law. In this study, the following maximum principal stress criterion is adopted as the basis of damage initiation.
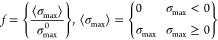
6Here, σ_max_^0^ is the critical maximum principal stress. *f* is the fracture criterion, and fracture will occur when *f* ≥ 1. The damage evolution law describes the stiffness degradation rate of materials. *D* represents the total damage to the material with an initial value of 0 and a total failure value of 1. The relationship between the stress component and damage variable *D* is as follows
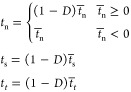
7

In this study, the energy BK (Benzeggagh-Kenane) criterion was used for simulations.

#### 2.4.2 Principle of the Extended Finite Element Method (XFEM)

As shown in Figure 3b, the extended finite element allows the crack to propagate through the element, which can easily simulate the crack propagation problem. The extended finite element approximates the crack as a point *x* in the finite element model and a number of discontinuous n nodes elements in any domain. Formula 8 is used to calculate the displacement of point *x* in the domain
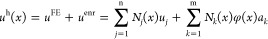
8where *u*^FE^ is the displacement field determined by conventional finite element approximation. *u*^enr^ is the enrichment function, considering any discontinuous existence. *N*_*j*_(*x*) and *N*_*k*_(*x*) are the shape functions of the finite element. *u*_*j*_ is the freedom vector of the conventional node. *a*_*k*_ is the set of extra degrees of freedom, and φ(*x*) is the discontinuous enrichment function.^44^

## 3 Results and Discussion

### 3.1 Tensile Behavior of Single Nanoparticle-Toughened Epoxy

Uniaxial tension and biaxial tension were applied to three kinds of single-nanoparticle/epoxy composites in which the particles are nano core–shell polymer, liquid rubber, and nanosilica, respectively. It is worth noting that in order to eliminate the influence of stress concentration in the boundary region in the uniaxial tension of a single-nanoparticle/epoxy composite, the average Mises stress is calculated without the boundary region.

#### 3.1.1 Uniaxial Tensile Properties

**Figure 4:**
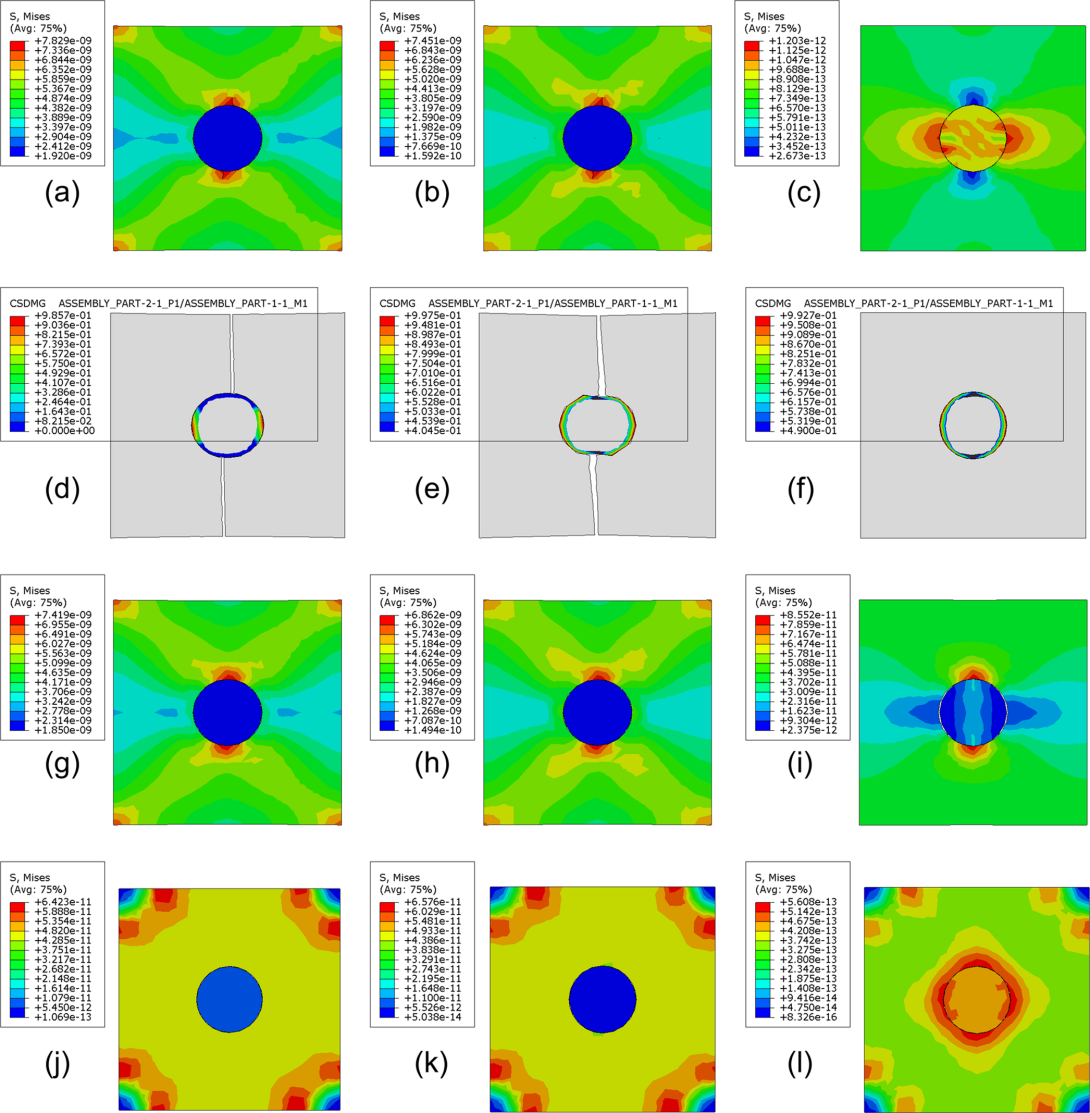
Tensile behavior of single nanoparticle-toughened epoxy. Stress envelope of (a) CSP and (b) LR models when a crack occurs. (c) Stress nephogram of the SI model at the beginning of loading. Interfacial crack diagram of (d) CSP, (e) LR, and (f) SI models when uniaxial tension is applied. Stress nephogram of the (g) CSP, (h) LR, and (i) SI models when the relative displacement is 0.1. Stress envelope of (j) CSP, (k) LR, and (l) SI models when biaxial tension begins.

When the boundary displacement of the CSP/epoxy single-particle model under uniaxial tensile load reaches 15 nm, cracks will occur at the two poles of the particle area (Figure 4a). Subsequently, the crack extends to the boundary along the direction perpendicular to the loading direction, and then the particles separate from the matrix with the application of tensile load. As shown in Figure 4d, the displacement of the loading boundary is 42 nm when the interface separation starts. During loading, when the relative boundary displacement is 0.1 (Figure 4g), the maximum and average Mises stresses are 74.19 and 33.63 MPa, respectively. The stress concentration factor is 2.21.

When a single LR particle-toughened system is applied with a uniaxial tensile load, its stress distribution is similar to that of the CSP model. The stress concentration also occurs at two poles of the particle area perpendicular to the loading direction when the boundary displacement reaches 13 nm, where crack initiation occurs, as shown in Figure 4b. However, due to the smaller modulus of liquid rubber particles, the particle–matrix interface separation will occur when the matrix is damaged to a greater extent than the nano core–shell polymer toughening system. At this time, the boundary displacement is 52 nm (Figure 4e). In addition, when the relative boundary displacement is 0.1, the maximum and average Mises stresses are 68.62 and 32.07 MPa, respectively, and the stress concentration factor is 2.14 (Figure 4h).

The stress distribution and failure process of the SI single particle-toughened system under uniaxial tension are quite different from those of the CSP and LR models. The main reason is that the modulus of nanosilica particles is high, and the interface performance with the epoxy resin matrix is poor. During the initial loading, stress concentration occurs at the two poles along the loading direction because Young’s modulus of nanosilica is far greater than that of the epoxy resin matrix (Figure 4c). Due to the low particle–matrix interface performance of the SI model, interface separation occurs rapidly at the two poles of the particle area along the loading direction when the loading continues, and the interface separation expands rapidly until the particles and the matrix are completely separated (Figure 4f). In addition, the lower interface performance results in a smaller relative displacement when interface separation occurs (0.6% for the SI model, 1.2% for the CSP model, and 1.3% for the LR model). The matrix and particles lose the load transfer ability, and the stress concentration area begins to transform to the two poles perpendicular to the loading direction after the interface separation occurs at the two poles along the loading direction around the particles. As shown in Figure 4i, when the relative boundary displacement is 0.1, the maximum and average Mises stresses are 85.52 and 27.30 MPa, respectively, and the stress concentration factor is 3.13. We can find that the stress concentration factor of the SI model is the largest, which will cause the SI model to break earlier than the other two models. The main reason is that the area around the particles along the loading direction forms pores, and the load cannot be transferred after the particle–matrix interface separation occurs, which makes the stress concentration in the area perpendicular to the loading direction more serious.

In a word, under uniaxial tensile load, the SI model is significantly different from the other two models due to its high particle modulus and low interface performance. On the one hand, interface separation occurs first, and then crack propagation occurs in the SI model, while crack propagation occurs first, and then interface separation occurs in the other two models. On the other hand, the stress concentration area of the SI model changes from the loading direction to the direction perpendicular to the loading direction during the loading process, while the other two models do not have this phenomenon.

#### 3.1.2 Biaxial Tensile Properties

The stress distribution of the single-particle CSP model and single-particle LR model under biaxial tensile load is similar, and the stress distribution near the particle area is relatively uniform (Figure 4j,k). There is still no interface separation when the loading boundary of the two models is seriously damaged. The stress distribution around the particle is relatively uniform, and there is no obvious stress concentration when biaxial tensile load is applied to the single-particle SI model, which is similar to the other two particle models. The difference with the other two kinds of particles is that due to the large modulus of nanosilica particles, the Mises stress around the particles is significantly higher than that in other areas (excluding the stress concentration area on the boundary), as shown in Figure 4l.

### 3.2 Effect of Nanoparticle Volume Content on the Mechanical Behavior of Toughened Epoxy

In order to study the influence of nanoparticle content on the tensile and fracture properties of epoxy resin, we established a series of multiparticle models (the particles are CSP, LR, and SI) with the volume fraction from 2.5% to 20% based on the model size and other parameters obtained above.

#### 3.2.1 Tensile Behavior

**Figure 5:**
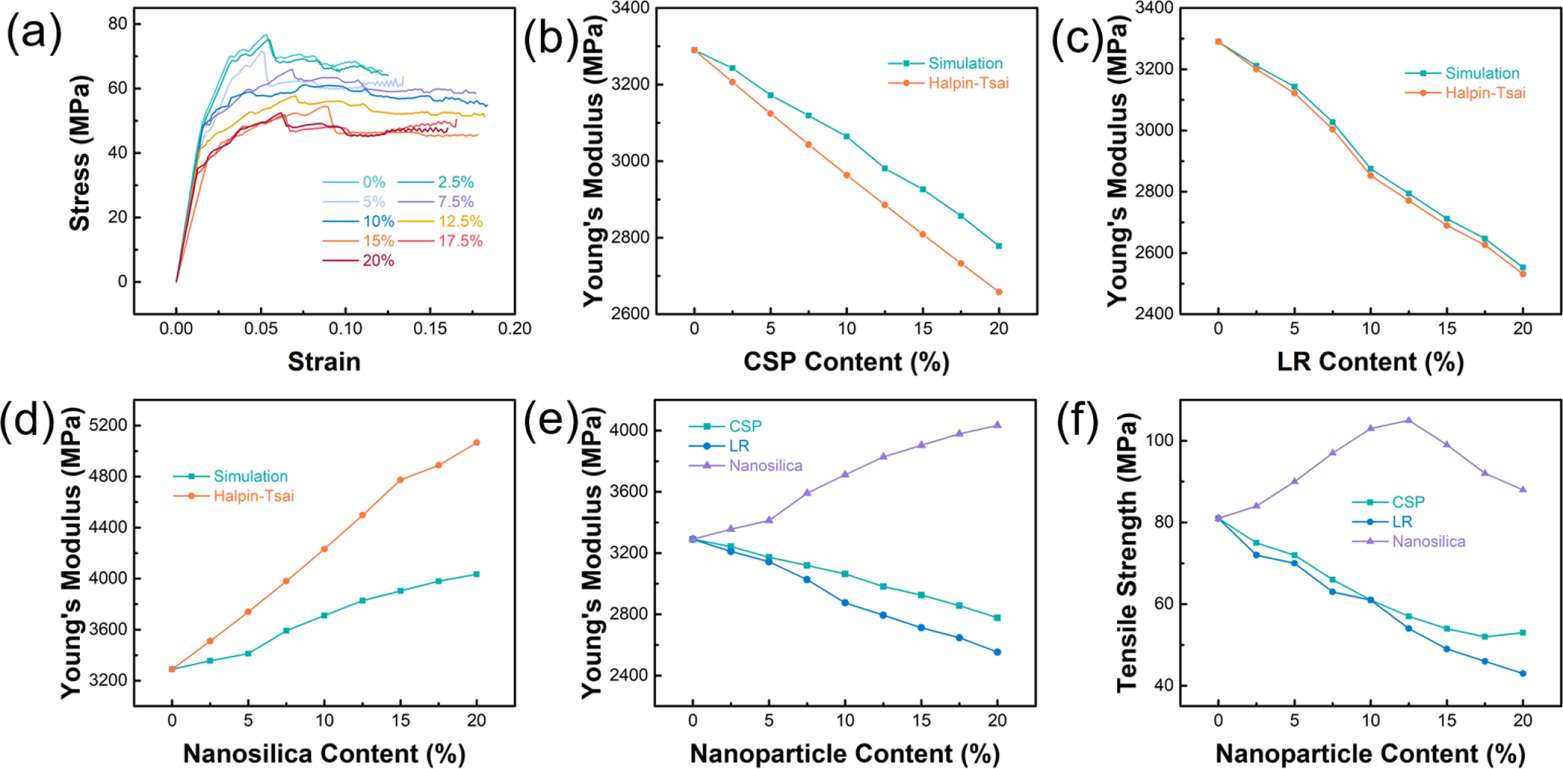
Effect of nanoparticle volume content on the tensile behavior of toughened epoxy. (a) Stress–strain curves of epoxy toughened by nanoparticles with different volume fractions. The simulation results compared with the Halpin–Tsai formula diagram of Young’s modulus of (b) CSP, (c) LR, and (d) SI models. Young’s modulus (e) and tensile strength (f) of different particle models under different nanoparticle contents.

Unidirectional tensile load is applied to the CSP model. We select the stress (S11) and strain (E11) along the loading direction of each element from the calculation results, then calculate the average value, and draw the stress–strain curve, as shown in Figure 5a. Young’s modulus and tensile strength of epoxy resin modified with different contents of CSP can be determined by the stress–strain curve and compared with the results calculated by the Halpin–Tsai semiempirical formula. As shown in Figure 5b, the variation trend of the finite element simulation results is consistent with that of the theoretical calculation results, which proves the correctness of this study. Young’s modulus (Figure 5b) and tensile strength (Figure 5f) of the modified epoxy resin decrease with the increase in CSP particle content. When the volume fraction of added CSP was 20%, Young’s modulus and tensile strength decreased by 15.6 and 34.6% compared with pure epoxy resin, respectively.

The simulation results of Young’s modulus and tensile strength of the LR toughened system are like those of the SI toughened system. Since the modulus of LR particles is much lower than that of CSP, Young’s modulus of LR toughened systems with the same volume fraction is lower. The simulation results are compared with those calculated by the Halpin–Tsai semiempirical formula, as shown in Figure 5c. Young’s modulus in the simulation results was higher than that calculated by the Halpin–Tsai semiempirical model. The simulation results show that the tensile strength decreases with the increase in the LR volume fraction (Figure 5f).

The method of calculating Young’s modulus and tensile strength of the SI toughened epoxy resin model is the same as that of the above two particles. The modulus of modified epoxy increases because the modulus of SI particles is larger than that of epoxy resin, showing the same trend as that calculated by the Halpin–Tsai formula. The simulation results are smaller than that of the formula, as shown in Figure 5d. According to the simulation results, the tensile strength of the modified epoxy resin first increases and then decreases when the volume fraction of SI reaches 12.5%, as shown in Figure 5f. SI particles in a certain concentration range can improve the tensile properties, which is an important advantage over CSP and LR toughening methods.

In this section, models of toughening particles using CSP, LR, and SI are established, and the relationship between tensile properties and the particle volume fraction is obtained. For CSP and LR models, both Young’s modulus and tensile strength decrease with the increase of particle volume fraction, and the decrease of the LR model is greater. The simulation results of the SI model are quite different from those of the first two kinds of particle models. With the increase of particle volume fraction, Young’s modulus increases, and tensile strength increases first and then decreases. The effects of the three kinds of particles on Young’s modulus and tensile strength of the modified epoxy resin are shown in Figure 5e,f. Therefore, adding SI particles is a reasonable choice if epoxy resins with better tensile properties are needed.

#### 3.2.2 Fracture Properties

**Figure 6:**
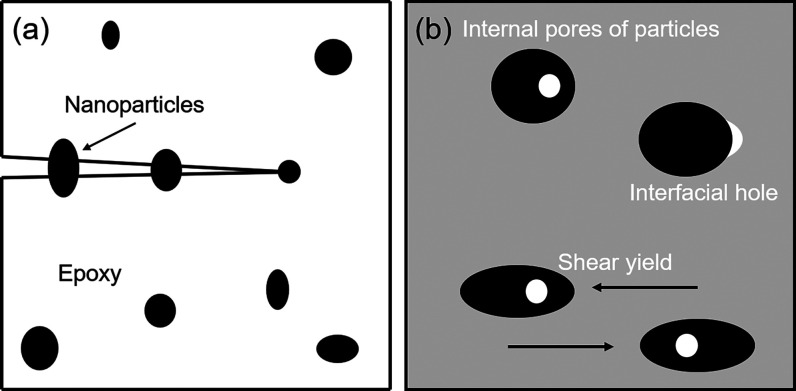
Mechanism of toughening epoxy by nanoparticles. (a) Anchoring mechanism. (b) Hole shear yield mechanism.

There are two main mechanisms for the epoxy resin toughened by nanoparticles: one is the anchoring mechanism (Figure 6a), and the other is the hole shear yield mechanism (Figure 6b). The anchoring mechanism refers to the introduction of foreign phase particles into the continuous epoxy phase. When microcracks are generated and extended in the epoxy phase, foreign phase particles act as bridges or anchors in the epoxy phase. Their elongation or tearing constrains the further expansion or extension of microcracks and prevents the formation of macroscopic fractures. In the hole shear yield mechanism, the external particles in epoxy resin are subjected to hydrostatic tension in the process of curing and cooling, and the front end of the crack is subjected to a three-directional stress field during loading. The combination of these two effects leads to the formation of holes inside the external particles or at the interface between the external particles and the matrix. On the one hand, these holes absorb energy. On the other hand, it induces the local shear yield between the foreign particles and the matrix resin, which leads to the passivation of the crack tip and prevents the occurrence of macroscopic fracture.

**Figure 7:**
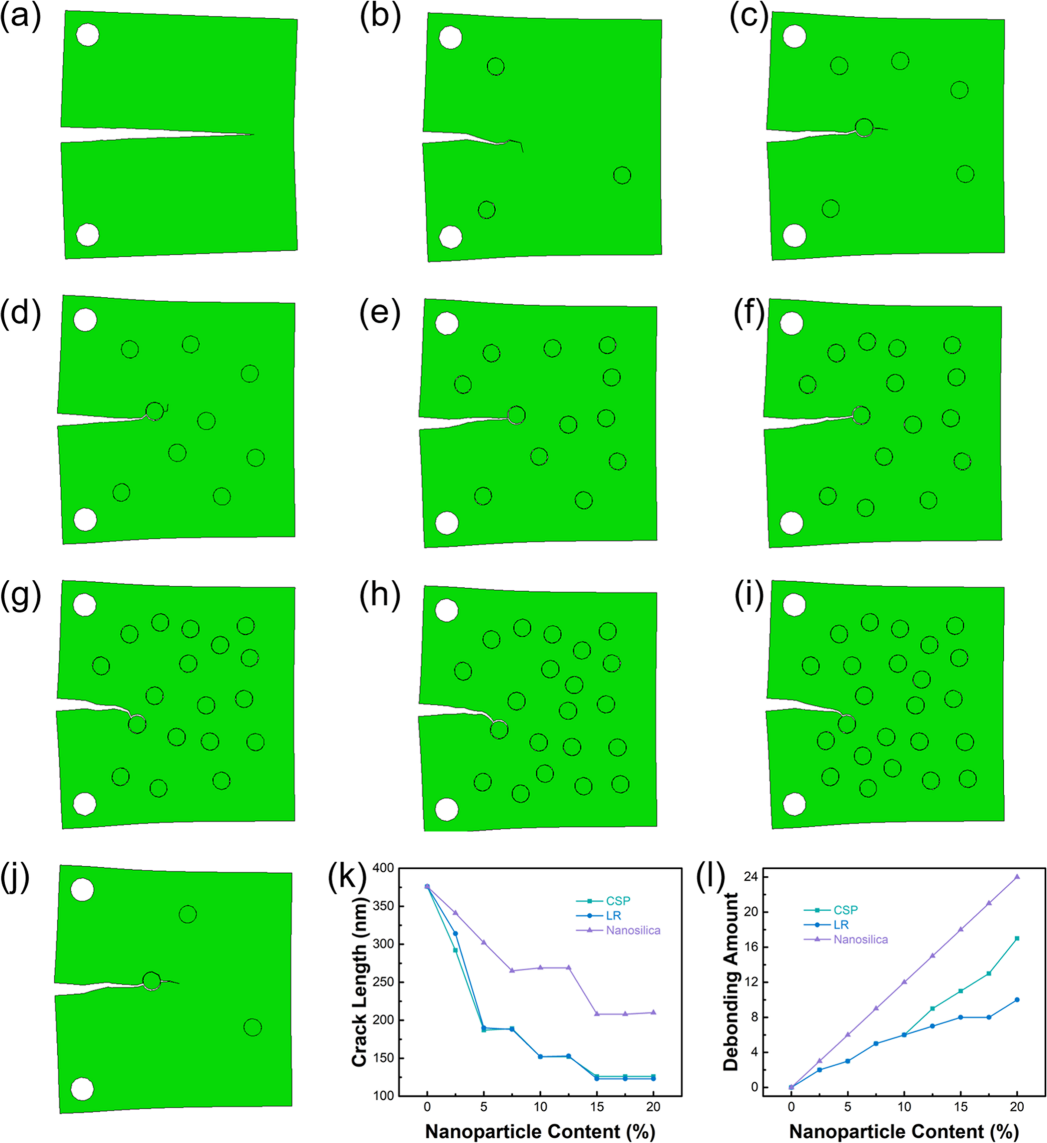
Effect of the nanoparticle volume content on the fracture properties of toughened epoxy. Crack growth in a toughened system of CSP with particle volume fractions of 0% (a), 2.5% (b), 5% (c), 7.5% (d), 10% (e), 12.5% (f), 15% (g), 17.5% (h), and 20% (i). (j) Another form of crack growth at a particle content of 2.5%. Crack length (k) and debonding amount (l) varying with particle content.

The crack growth of CSP toughened systems with different particle volume fractions is shown in Figure 7. For the epoxy resin without toughening particles, the crack propagated along a straight line with a length of 3760 nm. When the volume fraction of 2.5% CSP was added, the deflection to the stress concentration region appeared, and the length of crack propagation perpendicular to the loading direction was shortened to 2922 nm. When the volume fractions of CSP are 5 and 7.5%, the crack propagates through the particles, which shows the anchoring mechanism. When CSP with volume fractions of 10 and 12.5% was added, the crack propagation stopped in the particle region. It is speculated that the main reason is that the number of surrounding particles increases and that the interface separation formed holes consume part of the energy, which reflects the pore shear yield mechanism. When CSP with volume fractions of 15, 17.5, and 20% was added, crack growth stopped at a newly added particle closer to the preset crack tip.

The crack propagation of the LR toughened system is similar to that of CSP. The difference is that fewer particles are separated at the particle–matrix interface in the LR toughened system. The main reasons are inferred as follows: First, the small modulus of LR results in the stress around the interface being smaller under the same deformation degree. Second, the modulus of the epoxy resin modified by adding LR is smaller than that modified by adding CSP, so the stress of the model is smaller when it meets the displacement load requirements. Therefore, it can be predicted that in macroscopic materials, the shear deformation degree and energy absorption induced by CSP are larger, and the toughening effect should be better than that of LR under the same conditions.

The modulus of SI particles is large, and the interface property between SI particles and the matrix is low; therefore, many holes appear in the fracture process. These pores spread quickly, resulting in the complete separation of SI particles from the matrix. Because SI particles absorb less energy in the process of separation from the matrix and complete separation makes the anchoring effect of the particles unable to be reflected, the hindering effect of SI particles on crack propagation is not as good as CSP and LR. In addition, the high modulus of SI particles leads to the increase of the overall modulus of the modified epoxy, so the higher stress under the same displacement constraint leads to the larger crack propagation length in the simulation results.

It should be noted that in the microscopic model, the distribution of particles will significantly affect crack growth. For example, in the CSP toughened system with the same volume fraction of 2.5%, if the particle position is close to the initial crack, the crack will spread through the particle, and the propagation length will be significantly reduced (Figure 7j). According to the simulation results, the crack length and the number of particles with interface separation of different nanoparticle-toughened epoxy resins are shown in Figure 7k,l. The crack length presents a stepwise decline as the number of particles increases, which is related to the location of random nanoparticles. When the nanoparticles appear closer to the propagation path, the crack propagation length decreases significantly. In addition, the decrease of crack length is the result of the energy dissipation caused by the interfacial separation of nanoparticles and the matrix and the inhibition of crack tip extension by nanoparticles.

### 3.3 Tensile Properties of Epoxy Resins Toughened by Nanoparticles with Different Parameters

According to the different simulation results of the three kinds of particles, the main factors affecting the crack propagation in the microscopic model are the modulus of particles and the interfacial properties of particles and the matrix. The single-particle model and the multiparticle model with different volume fractions were selected to simulate the control variables of these two factors, respectively. The critical principal stress in the damage initiation criterion is used to characterize the particle–matrix interface performance.

#### 3.3.1 Single-Particle Model Simulation of Toughened Particles with Different Properties

The modulus of the epoxy matrix is generally around 3 GPa. Therefore, particles whose modulus is higher than that of epoxy are referred to as high-modulus particles in this paper, and nanoparticles whose modulus is 10 and 100 GPa are simulated. The particles whose modulus is lower than that of epoxy are called low-modulus particles, and the nanoparticles whose modulus is 1 GPa, 100 MPa, and 10 MPa are simulated.

For high-modulus particles, the following situations appear successively with the decline of the interface properties.

**Figure 8:**
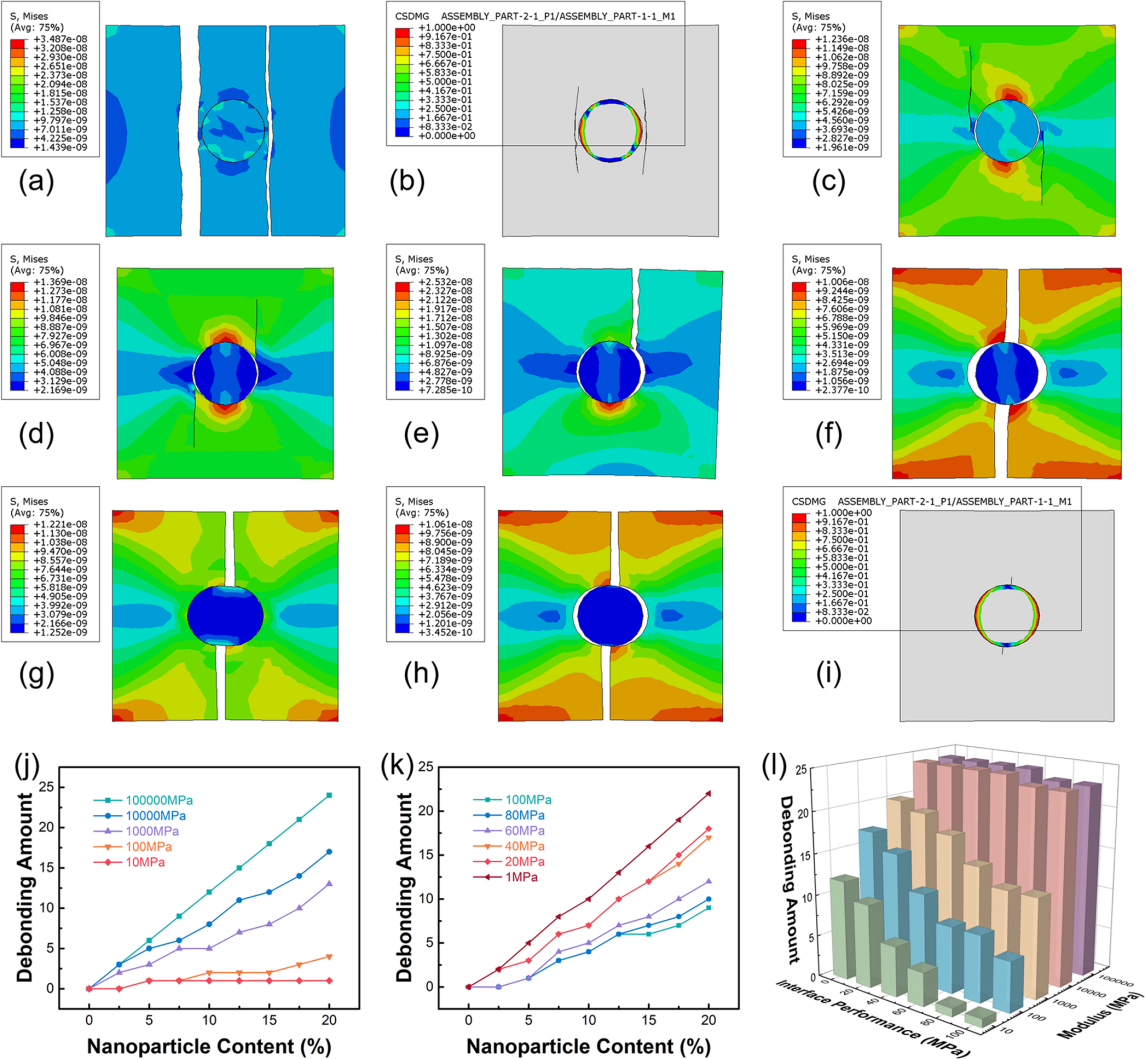
Tensile properties of epoxy resins toughened by nanoparticles with different parameters. (a) Cracks appear on both sides of the particle, and the interface is not separated. (b) Cracks appear on both sides of the particle, and then the interface separates. (c) Interfacial separation followed by cracks on both sides of the particle. (d) The crack moves toward the central region of the particle. (e) Cracks appeared on one side of the particle. (f) Cracks appear at two poles perpendicular to the loading direction. (g) The interface is not separated when the matrix is damaged greatly. (h) The interface was separated after cracks appeared at the two poles perpendicular to the loading direction. (i) Cracks appear at two poles perpendicular to the loading direction after interface separation. (j) The relationship between the number of particles separated at the interface and the volume fraction is generated for toughened particles with different modulus (the interface performance is 50 MPa). (k) The relationship between the number of particles separated by the interface and the volume fraction is generated for toughened particles with different particle–matrix interface properties (the particle modulus is 1 GPa). (l) Number of particles separated at the interface under different particle moduli and interface properties.

(1) Cracks appear on both sides of the particle along the loading direction, and the interface is still not separated when the damage degree of the matrix is very serious (Figure 8a).
(2) After cracks appear on both sides of the loading direction, the interface is separated (Figure 8b).
(3) Interface separation occurs first, and then cracks appear on both sides of the particle along the loading direction (Figure 8c).
(4) Interface separation occurs first, and the crack region moves from the two sides of the particle along the loading direction to the two sides of the particle perpendicular to the loading direction (Figure 8d).
(5) Interface separation occurs first, and cracks appear in the unilateral region perpendicular to the loading direction of the particles (Figure 8e).
(6) Interface separation occurs first, and two cracks appear at two poles perpendicular to the loading direction of the particle (Figure 8f).

The critical interface properties of these six conditions when the particle modulus is high are listed in Table 5. Although the particle modulus is 10 times different, the maximum stress of critical interface separation has little difference. When the particle modulus reaches more than 10 GPa, it has little effect on the stress size and distribution during failure. Therefore, the failure mode of epoxy resin composites toughened by high-modulus particles is mainly determined by particle–matrix interface properties.

**Table 5:** Critical Interface Properties of Six Conditions When the Particle Modulus is High (Unit: MPa)

particle modulus	(1)	(2)	(3)	(4)	(5)	(6)
100000	>78	54–78	50–54	13–50	11–13	<11
10000	>76	54–76	47–54	12–47	10–12	<10

In the case of a low modulus, the following situations occur successively with the decline of interface performance.

(1) Cracks appear at two poles perpendicular to the loading direction of the particle, and the interface is still not separated when the damage degree of the matrix is very serious (Figure 8g).
(2) The interface is separated after the crack occurs at the particle perpendicular to the loading direction pole (Figure 8h).
(3) Interface separation occurs first, and then cracks are generated at the particle perpendicular to the loading direction pole (Figure 8i).

The critical interface properties of these three cases when the particle modulus is low are listed in Table 6. When the modulus of the particle is low, its change also affects the failure form. Therefore, the failure form of toughened epoxy with low-modulus particles is determined by both the modulus and the interface properties.

**Table 6:** Critical Interface Properties of Three Conditions When the Particle Modulus is Low (Unit: MPa)

particle modulus	(1)	(2)	(3)
1000	>208	22–208	<22
100	>29	3.2–29	<3.2
10	>3.5	0.4–3.5	<0.4

#### 3.3.2 Effect of Toughened Particle Properties on the Number of Particles with Interfacial Separation

In the two toughening mechanisms of nanoparticles, the particle–matrix interface separation is an important reason to prevent crack growth. Studying the effect of particle properties on the number of particles with interfacial separation during fracture can better determine the failure process of a modified epoxy resin. By setting different modulus and interface performance parameters for the multiparticle model with a gradient increase in volume fraction, the number of particles with interfacial separation in the multiparticle toughening system with different performances and volume fractions can be obtained.

According to the simulation results, the higher the particle modulus, the more the particles are separated at the interface (Figure 8j). The lower performance of the particle–matrix interface results in a greater number of particles with interfacial separation (Figure 8k). The number of particles with interface separation corresponding to different particle moduli and interface properties is shown in Figure 8l. It is worth mentioning that the number of particles with interfacial separation is not directly positive or negative with a correlation with the effect of preventing crack growth. The effect of preventing crack growth is also related to the energy consumed in the process of interfacial separation and the range of shear plastic zone caused. This view can be verified in the conclusion obtained in Section 3.2.2. Although SI has the largest number of particles separated, its toughening effect is the worst.

## 4 Conclusions

In this paper, the mechanical behavior of epoxy toughened by a nano core–shell polymer, liquid rubber, and nanosilica was studied by simulations. The following conclusions are obtained from this study.

(1) In the single-nanoparticle model, uniaxial tensile load is more conducive to the formation of cracks. Cracks occur first in CSP and LR models, and interface failure occurs first in the SI model.
(2) The addition of CSP and LR will reduce the tensile properties of epoxy resin, and the addition of appropriate SI will improve the tensile properties of epoxy resin.
(3) The crack propagation length decreases with the increase of the content of nanoparticles and presents a stepwise decline.
(4) The toughening effect of the nano core–shell polymer is the best.
(5) When the modulus of toughened particles is greater than that of epoxy resin, the failure mode is determined by the properties of the particle–matrix interface. When the modulus of toughened particles is less than that of epoxy resin, the failure mode is determined by the properties of the particle–matrix interface and the particle modulus.

The numerical simulation model used in this project fills the research blank related to nano toughening and provides a reference for us to select nano toughening particles. Other similar nanoparticle toughening simulations can also be performed in the future by this method. But there is still a gap with the actual materials. The particle agglomeration phenomenon is not considered in determining the particle distribution. In the fields of chemical engineering, materials, and so on, how to study the formation and influence of particle agglomeration in microscopic models is a problem worth studying in the future. In addition, this study did not consider the toughening effects of other shaped nanofillers such as nanofibers and nanoplatelets, and the research methods of related contents need to be further explored in the future.
